# Water security determines social attitudes about dams and reservoirs in South Europe

**DOI:** 10.1038/s41598-022-10170-7

**Published:** 2022-04-12

**Authors:** Eduardo Dopico, Elena Arboleya, Sara Fernandez, Yaisel Borrell, Sonia Consuegra, Carlos García de Leaniz, Gloria Lázaro, César Rodríguez, Eva Garcia-Vazquez

**Affiliations:** 1 Department of Education Sciences, University of Oviedo, Oviedo, Asturias Spain; 2 Present Address: Department of Functional Biology, University of Oviedo, Oviedo, Asturias Spain; 3 Department of Biosciences, Swansea University, Swansea, UK; 4 AEMS-Ríos Con Vida, Oviedo, Spain

**Keywords:** Ecology, Environmental social sciences

## Abstract

River barriers affect river dynamics and aquatic biota, altering the entire ecosystem. Nevertheless, dams and reservoirs provide goods like water supply and low-carbon energy that are becoming increasingly critical under current climate change. To know to what extent dams and reservoirs are important to the population, we explored social attitudes towards dams and reservoirs using a face-to-face questionnaire in two regions of contrasting climate and water security in Spain, a country with one of highest densities of dams in Europe. Results (N = 613) revealed a higher support for dams, mediated by the recognition of the services they provide, in the drier Mediterranean Malaga province (Andalusia), than in the wetter Atlantic Asturias province (Bay of Biscay), where water shortages are rare. Awareness of the impacts of the dams was more pronounced in Malaga, coupled with a higher willingness to pay for reconnecting rivers. Social awareness of both impacts and services provided by dams and reservoirs may depend on local climate and water security; different dam acceptance emphasizes the need to involve local citizens in the decision-making processes about water management.

## Introduction

Human history is linked to the riverbeds. Rivers cover only a small area of the planet but are vital for human survival and economy; their drinking water, fishing resources and navigation facilitated the first human settlements. Although they are part of our cultural heritage, rivers are often managed as a political or engineering challenge^1^. Large dams and reservoirs are constructed to obtain energy and water supply, but cause enormous environmental impacts, contributing to increase greenhouse gases emissions^2^, especially during construction^3^. They disrupt the movement of sediments and nutrients^4^. However, water infrastructures like dams are essential to achieve Sustainable Development Goals because they guarantee water security today^5^.

Since rivers are crucial for the planet’s water cycle, the EU Water Framework Directive 2000/60 (Official Journal of the European Communities, 327, 22.12.2000, pp. 1–73), requires Members States to achieve good ecological status or good potential for all water bodies, and includes the reduction of environmental impacts caused by artificial barriers. Unfortunately, just under half of the surface waters of the EU met the good ecological status target of the 2015 Water Framework Directive^6^. The density of river barriers is enormous in Europe^7^. The main rivers of Europe are disconnected from the sea and this has had a catastrophic impact on many species^8^ including some emblematic migratory fish such as salmon, sturgeon or eel that, in some cases, have been driven to extinction. Dams affect the physical–chemical quality of rivers^9^, alter the natural flow regimes^10^ and modify sediment transport and water temperature^11,12^. In addition to causing hydrological alterations, river barriers induce irreversible effects on river dynamics and aquatic biota, especially on fish^13^, by restricting their migratory movements and reproductive displacements. Altering natural biodiversity^14,15^, colonization by invasive species is favored^16,17^^.^

One of the biggest challenges for achieving a good ecological status of rivers is restoration of connectivity. Building fish passages may restore upstream migration^18^, but does not render the river to its original condition. Movements like Dam Removal Europe group (https://damremoval.eu/, accessed September 2021) claim for dam demolition to set the rivers free, targeting especially obsolete dams, yet scientists recommend to considering carefully the potential environment impacts of demolitions^19^. This seems naïve when considering the conflicts that this perspective raises. Recognizing the negative effects of dams and reservoirs on intact ecosystems does not mean denying their benefits to provide energy, water supply and leisure opportunities. The current society cannot be conceived without the well-being associated with drinking-water supply and renewable energy supply for electricity in homes, workplaces and industries. Moreover, Benjankar et al.^20^ suggest that ecological benefits of dams may compensate their impacts, for example maintaining suitable habitats for vulnerable species during droughts. Measures that minimize the social^21^ and environmental impacts of dams must be found^8^; restoration costs are known to be generally high and are not balanced by a reasonable assessment of environmental costs^14^.

**Figure 1:**
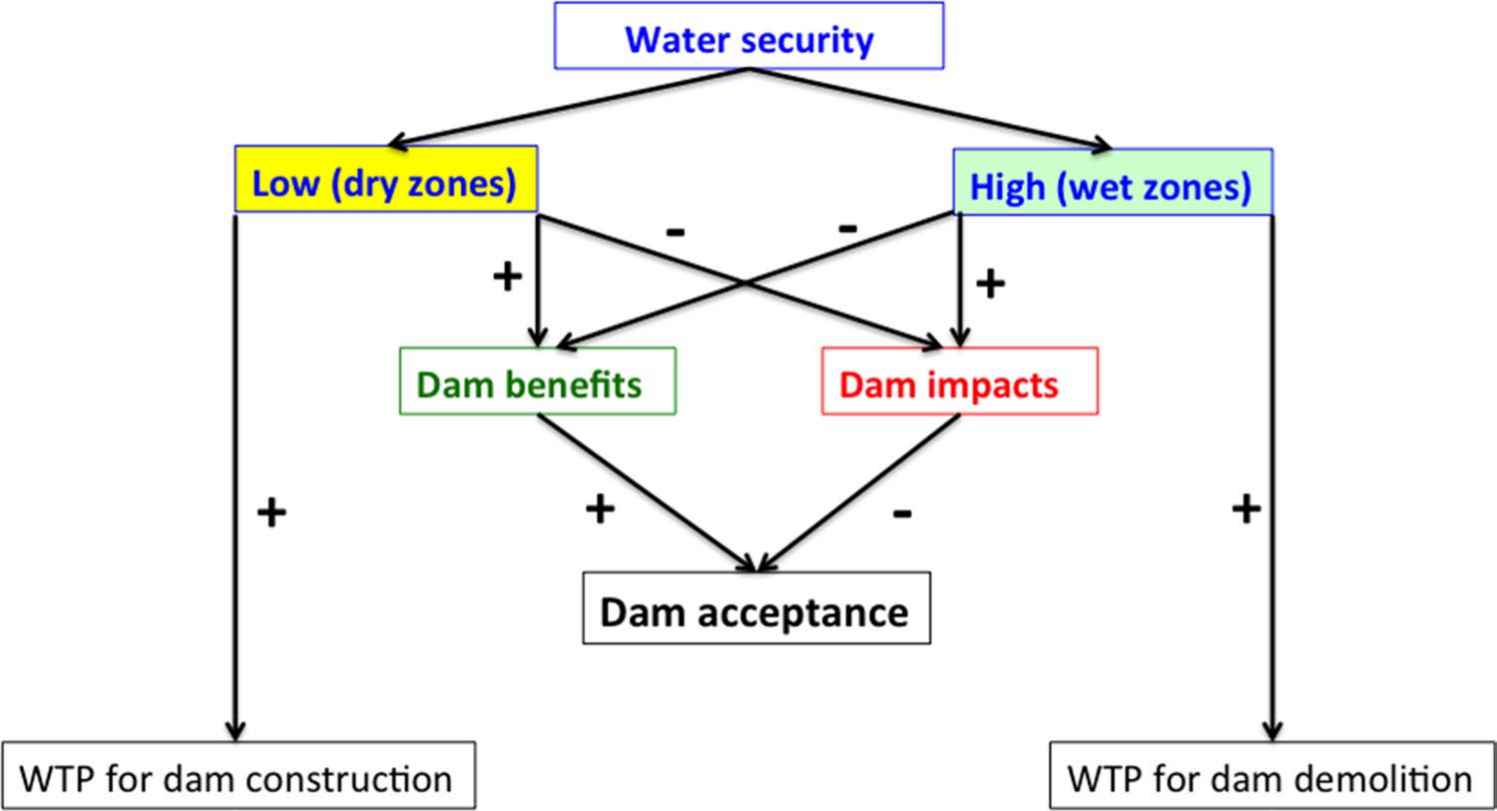
Visual summary of the departure hypotheses of this study.

Since humans depend on rivers for water, energy, food and leisure, raising awareness of their value is necessary for encouraging their protection^22^. Socio-political acceptance of energy policies at the European international and national level is sought to initiate hydropower projects^23^. While water storage structures are necessary for adjusting to climate change, their development is controversial and may generate opposition not only because of their ecological impact, but also because of the displacement of people and the destruction of livelihoods, making the investigation of the social acceptability of dams a priority^24,25^. Taking into account social attitudes towards dams and reservoirs is especially important for designing effective restoration strategies that are often guided by economic and social opportunism^26^. Research on dams, reservoirs and barriers should be made from an adaptive management perspective. That is, thinking that ecological resources are dynamic in nature and therefore their management and conservation must be adapted to the needs of the environmental context^27^. Measuring the degree to which citizens believe that those infrastructures are (or are not) beneficial for their activities and for the environment is therefore important. However, Yousefi-Sazhabi et al.^28^ found a research gap in the public awareness and social acceptance of renewable energy technologies like hydropower. Project sentiment analysis using social media demonstrated that almost half of the messages collected were negative about the large Three Gorges project dam in China^29^. In Brazil, the majority of participants in a household survey preferred leaving some free-flowing rivers while concentrating dam construction on others^30^. These authors also found that ecologically-oriented water policies were preferred by participants with ecological water values (Fig. 1).

**Figure 2:**
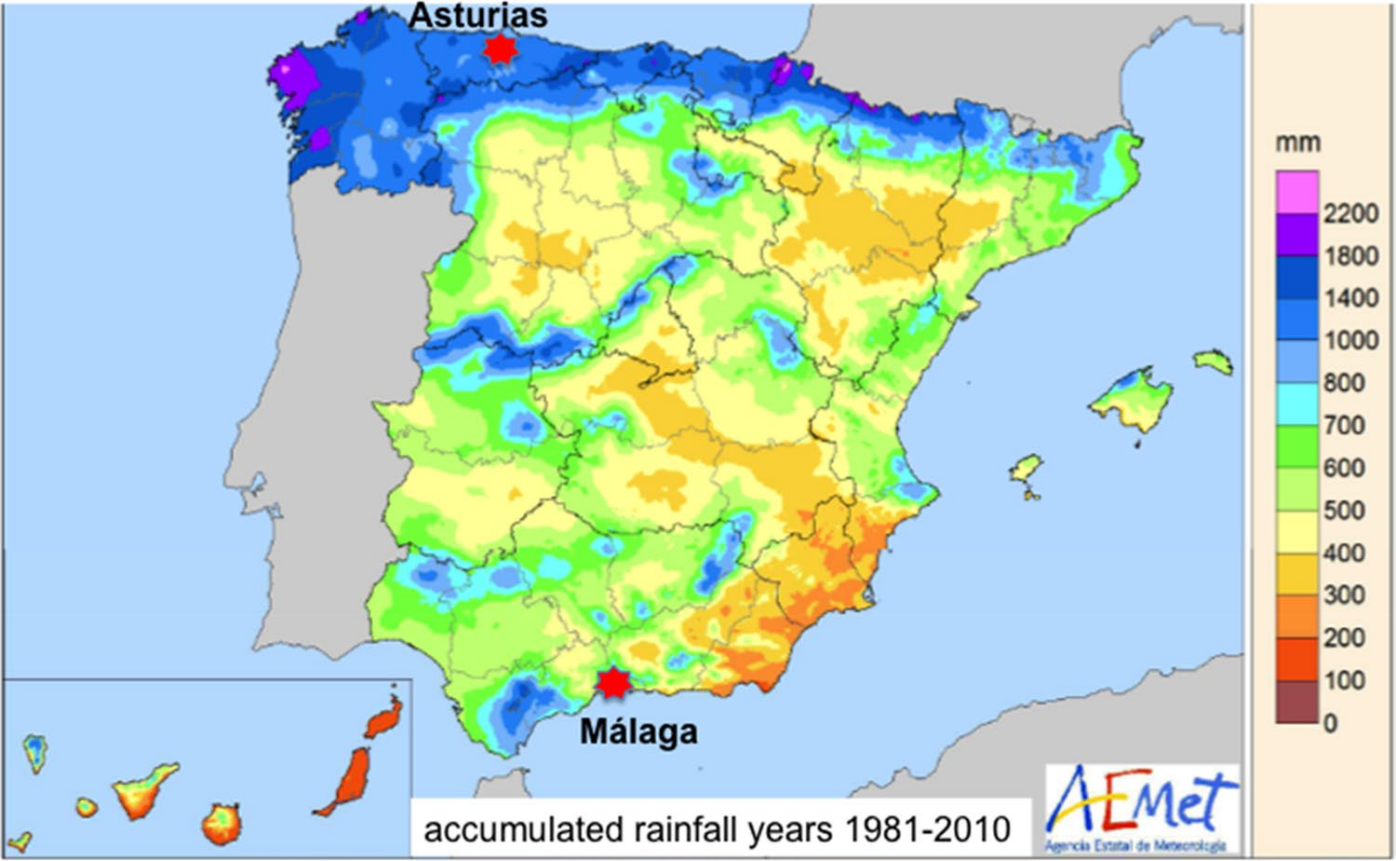
Map of Spain with sites where the study took place, showing mean annual rainfall between 1981 and 2010. Modified from the Open Data of the AEMET, State Meteorological Agency, Spanish Ministry for Ecological Transition, 2018. URL https://www.aemet.es/documentos/es/conocermas/recursos_en_linea/publicaciones_y_estudios/publicaciones/MapasclimaticosdeEspana19812010/MapasclimaticosdeEspana19812010.pdf (pp. 34). These documents and all their contents can be reproduced freely under the Open Access policy of AEMET (https://www.aemet.es/es/datos_abiertos/estadisticas/balance_hidrico).

The majority of studies about social impacts of dams are focused on populations affected by large dams in Asia, followed at a distance by Africa and Americas studies, then Europe in the last place^31^. Despite their importance for water and energy supply, their large number in European rivers and their recognized ecological impacts, few studies have addressed the social acceptance of dams and reservoirs in Europe by the general population. Using in-depth interviews (N = 153), Wiejaczka et al.^25^ found that people directly affected by a reservoir in Poland (i.e. displaced from their homes) perceived higher negative environmental impacts of dams than non-resettled people. They also found some gender bias; for example, resettled women perceived significantly more dam benefits than men^25^. Piróg et al.^32^ found in a sample of households affected by a Carpathian reservoir (N = 96) that dams acceptance is correlated with their perceived benefits, and with the sense of security. In a survey amongst Spanish university students (N = 964), Arboleya et al.^33^ showed that river connectivity was one of the least valued river features, water quality being considered the highest. There is a need for more representative samples of river users, targeting wider population sectors in Europe. To fill the gap, we developed a questionnaire about social attitudes towards dams and reservoirs, and interviewed people living in two regions with contrasting climate and water security, i.e. wet and dry areas in Spain, one of the countries with the highest density of dams in Europe^7^. The regions were the warm dry Malaga province (Mediterranean façade of Andalusia) and the colder, wetter province of Asturias (Bay of Biscay) where rainfall is high (Fig. 2, Table 1) and there are no water shortages. The number of dams is similar in the two provinces (Table 1). Expectation was that a higher dependence on dams for water security in dry regions will increase the perception of dam service provision, and decrease that of dam impacts; as a result, the acceptance of dams will be emphasized in dry versus wet provinces.

**Table 1:** Climate indicators and characteristics of dams in the two study regions.

		Asturias	Malaga
**Rainfall 2012/2017**		**73/190**	**12.1/12.5**
**Mean daily temperature**		**10.7/10.0**	**13.7/13.0**
**Number of dams**		**23**	**27**
**Mean construction year (SD)**		**1963.7 (21.8)**	**1966.3 (39.5)**
% use	Hydropower	56.5%	22.2%
	Drinking water supply	21.7%	22.2%
	Irrigation	0%	22.2%
	Water transfer	0%	29.6%
	Recreation	13.1%	7.4%
	Industrial	13.1%	0%
	Regulation	4.3%	0%

Total rainfall (L/m^2^) and mean daily temperature in December of 2012 and 2017. Number, construction year (mean in the region and standard deviation SD), and uses of dams presented as % of dams in a region for each use.

Our departure hypotheses, summarized in Fig. 1, can be formulated as:

1. Given the importance of dams for water security^5^, we expected a greater acceptance and positive attitudes towards dams in the drier Malaga than in the wetter Asturias province.
2. Since ecological awareness determines ecologically-oriented water policies^30^, the relationship between water security and dam acceptance would be negatively mediated by the perception of dam impacts.
3. For the correlation between perceived benefits and dam acceptance^32^, the relationship between water security and dam acceptance would be positively mediated by the perception of services provided by dams.
4. Willingness to pay for the construction of more dams will be higher in Malaga while, if perceived more negatively as expected in i), Asturias people would pay more for dam demolition.

## Results

To test the departure hypotheses, we constructed a questionnaire (Supplementary table 1) and validated it in a pilot survey, then administered it face to face to adult residents from Asturias and Malaga regions. The socio-demographic characteristics of samples were quite similar in the two regions (Table 2), with similar gender ratios (not significantly different; contingency chi-square = 0.19 with 1 d.f. and *P* = 0.67 > 0.05, ns) and education levels (chi-square = 1.59 with 2 d.f. and *P* = 0.45, ns). This is important because we could discard possible biases due to these socio-demographic variables that are related with environmental awareness in other studies, where females^25^ and persons longer involved in formal education^34^ are generally more aware about water issues.

**Table 2:** Sociodemographic characteristics of the survey participants in Asturias and Malaga.

	Asturias_ N = 296	Malaga_ N = 317
Gender (%females)	59.3	57.7
**Age group**		
< 30	42.3	31.2
30–60	47.7	57.4
> 60	10.0	11.4
**Education level**		
Primary	16.8	16.4
Secondary	16.7	20.8
Higher education	66.5	62.8
**Occupation sector**		
Agriculture	0.7	0.4
Industry	1.7	0.0
Building trade	1.6	0.0
Services	55.7	67.3
Unemployed	4.7	3.1
Retired	7.3	6.6
Homemaking	4.0	3.1
Student	24.3	19.5

### Questionnaire validation

In the pilot survey (N = 50), internal consistency was good for the services provided by dams and reservoirs Q2 (alpha = 0.861) and the perceived impacts Q4 (alpha = 0.85), and acceptable for the WTP Q3 (alpha = 0.742 after transforming tax increases in their respective Likert values). Test–retest results were highly significant: r = 0.876 with p < 0.001, r = 0.735 with *P* = 0.015 and 0.833 with *P* = 0.003 for whole test score, non-distracting items and distractors respectively. From the test–retest it was thus concluded that the survey was reliable. Further triangulation confirmed the validity of the questionnaire design.

In the regional surveys, significant multicollinearity could be discarded from low variable inflation factors between 1.001 and 1.21. The correlation between the first and the last answers to Q1 was positive and statistically significant both in Asturias (r = 0.689, 294 d.f., *p* < 0.001) and in Malaga (r = 0.698, 315 d.f., *p* < 0.001). This further confirmed that the design was valid.

### Difference between regions in perception and acceptance of dams

**Figure 3:**
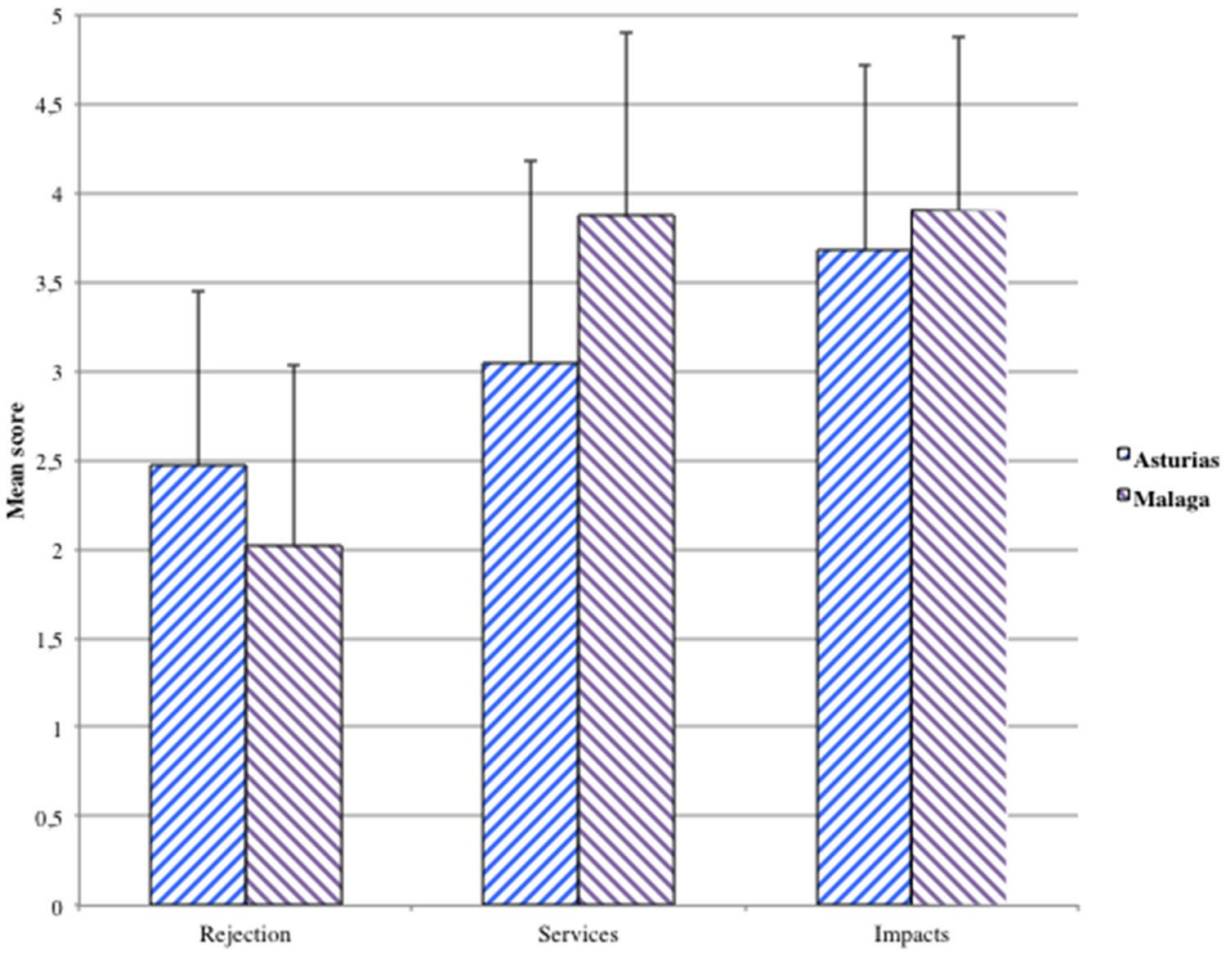
Public opinion about dams and reservoirs in Asturias (N = 296) and Malaga (N = 317), showing mean values of dam acceptance (left), impacts of dams (center) and benefits of dams (right) Results are presented as mean scores of each composite variable (construct), with standard deviation as capped bars.

The hypothesis about a higher acceptance (= lower rejection, the variable measured in our study) of dams in regions with lower natural water security (thus more dependent on dams for this purpose) was confirmed in our study. The results in the two regions with contrasting climate and water security indicated that respondents were more favorable to dams in the dry Malaga (N = 317) than in the wet Asturias (N = 296) (Fig. 3). The two-way ANOVA (Table 3) confirmed the difference between regions was highly significant (F(1, 1832) = 17.8 with *p* << 0.001). In Asturias the mean score for the rejection of dams (Q1) was a little bit smaller than 2.5 while it was 2 in Malaga (t = 5.51, *p* << 0.001). The benefits (services) provided by dams (central columns in Fig. 3) were more appreciated in Malaga than in Asturias (mean 3.85 versus 3.05 respectively, t = 9.42, *p* << 0.001). Interestingly, a higher appreciation of dams and dam services in Malaga did not imply ignoring their impacts. Unlike expectations, dam impacts were more strongly perceived in Malaga (mean of 3.9) than in Asturias (mean 3.7); t = 2.75, *P* = 0.006) (columns at right in Fig. 3).

**Table 3:** Two-way ANOVA testing the differences in mean values of the constructs measuring public opinion about dams and reservoirs in Asturias and Malaga.

Factor	Sum of squares	df	Mean square	F	p (same)
Region:	18.887	1	18.887	17.77	2.62E−05
Measure of dam perception:	830.686	2	415.343	390.7	4.80E−142
Interaction:	123.681	2	61.841	58.17	3.21E−25
Within:	1947.59	1832	1.063		
Total:	2920.71	1837			

Regarding the different measures of dams appreciation (representing different aspects of the public opinion about dams and reservoirs i.e. acceptance, impacts perceived and benefits perceived), they were also highly significantly different to each other (F(2, 1832) = 390.7 with *p* << 0.001, Table 3). The score of rejection was the lowest of the three measures in the two regions (Fig. 3), while the score of impacts was higher than (in Asturias) or equal to (Malaga) the score of services. Accordingly, the interaction between measures of dam perception and the regions was statistically significant as well (F(2, 1832) = 58.2, *p* << 0.001).

### Mediators between water security and dam acceptance

**Figure 4:**
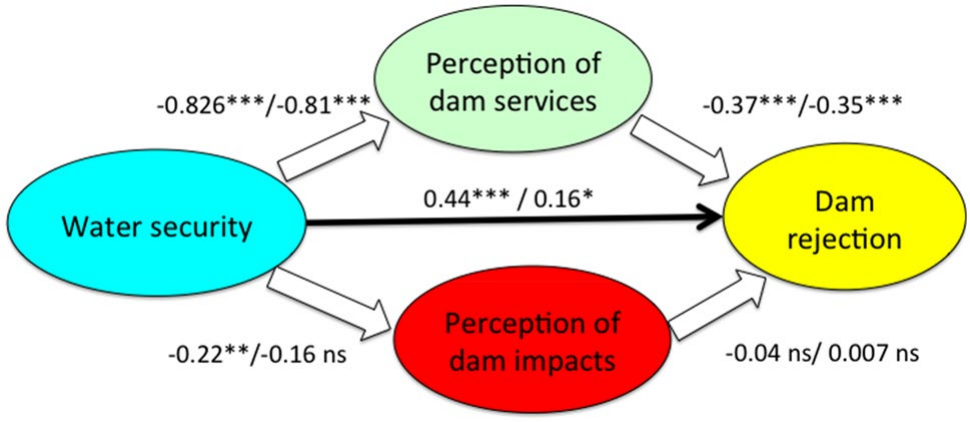
Mediation model proposed in this study, presenting β coefficients of the multiple regression model associated to arrows that represent relations between the variables involved. β values are presented as [total/partial after holding constant the other variables]. Not significant, ns; *p* < .05, *; *p* < .01, **; *p* < .001, ***.

Perceived dam services and impacts were expected to mediate between water security and dam acceptance (rejection in this study). This expectation was only partially confirmed from the multiple regression analysis (Table 4). On the one hand, the prediction of dam rejection from water security was less significant when holding dam services and impacts constant, which indicates a mediation effect (Fig. 4). Dam services predicted significantly dam rejection holding constant the rest of variables, thus could be considered significant mediators between water security and dam rejection (or acceptance).

**Table 4:** Results of the multiple regression analysis to test mediation effects of perceived services and impacts of dams. Dependent variable: dam rejection. SE: standard error.

	Coefficient	SE	t	*p*	r^2^
Constant	3.33	0.19	16.84	< .001	
Water security	0.16	0.08	1.99	.046	0.047
Dam services	−0.345	0.03	9.95	< .001	0.176
Dam impacts	0.007	0.04	0.19	.848	0.002

On the other hand, opposite to our expectation, the perceived dam impact was not a significant predictor of dam rejection holding other variables constant (Table 4, Fig. 3), thus it could not be a mediator between water security and dam rejection. The lack of importance of perceived impacts to determine dam acceptance in both Asturias and Malaga was supported from non-significant pairwise correlations (Table 5), while perceived services were indeed negatively correlated with dam rejection (r = −0.454 in Asturias and r = −0.296 in Malaga, both with *p* < 0.001; Table 5). Moreover, services and impacts were not significantly correlated in any region (Table 5).

**Table 5:** Pairwise correlations between the three constructs measuring opinions about dams and reservoirs in Asturias (below diagonal) and Malaga (above), Spain.

	Rejection	Services	Impacts
Rejection		−0.296***	−0.083
Services perceived	−0.454 ***		0.099
Impacts perceived	0.045	0.047	

***p < 0.001.

### Willingness to pay for actions on dams

**Figure 5:**
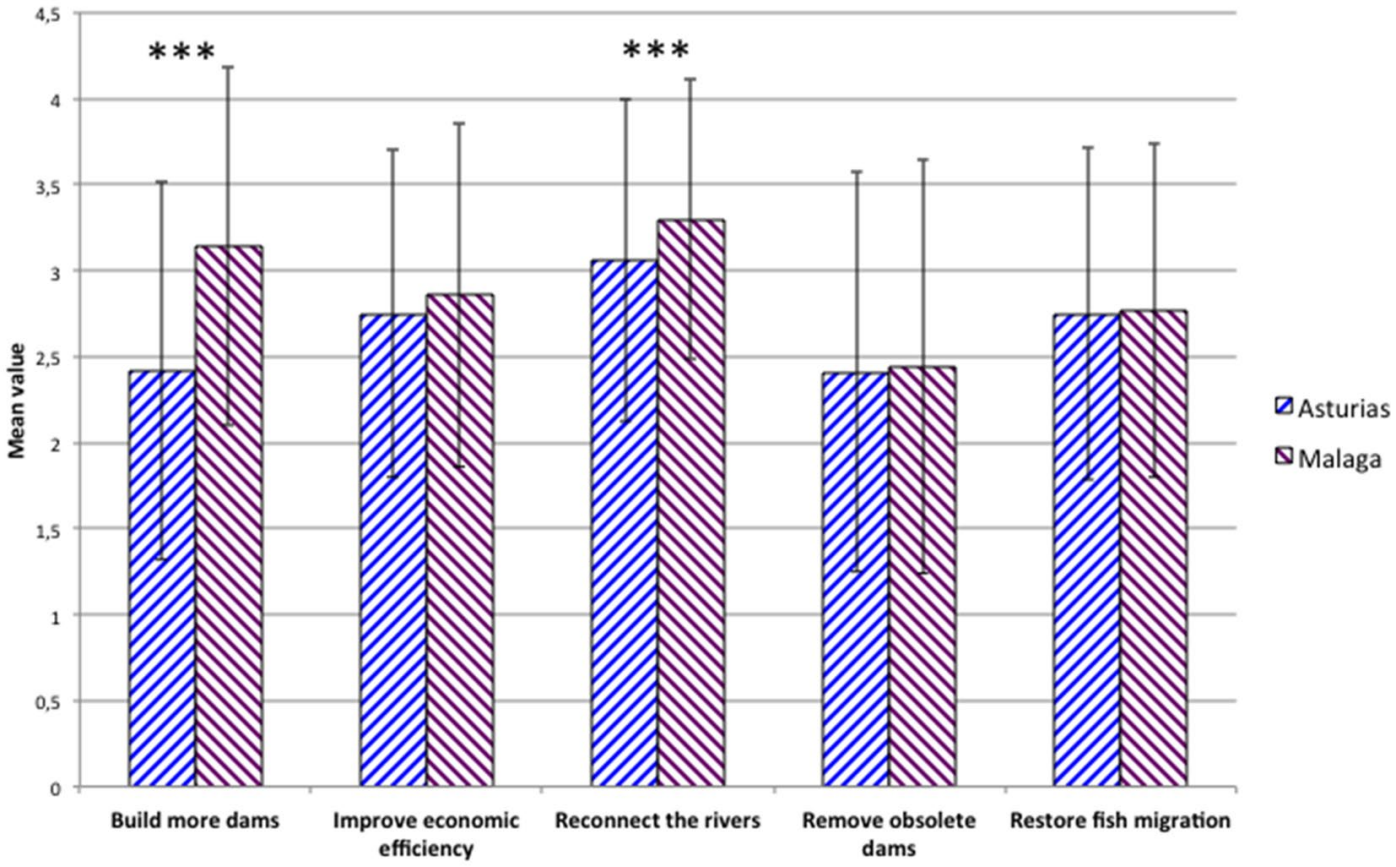
Willingness to pay for actions to improve dams and reservoirs services in Asturias and Malaga. Results are mean scores assigned to each action, being 1 = 0%, 2 = 0.1–0.5%, 3 = 0.5–1%, 4 = 1–5% and 5 = a higher % of tax increases. Standard deviation as capped bars. *** = p < .001 in t-test for the difference between Asturias and Malaga.

Willingness to pay **(**WTP) analysis revealed similar but not identical results in the two regions. We can see in Fig. 5 the mean scores assigned to five actions to improve dams’ ecosystem services (or disservices) in Asturias and Malaga. Reconnecting the rivers to improve connectivity was the most valued action and remove obsolete dams the least one in the two regions. However, participants from Malaga would pay significantly more to reconnect the rivers than those interviewed in Asturias (mean value in 1–5 Likert scale of 3.3, SD 0.82 in Malaga versus 3.06, SD 0.94 in Asturias; t = 3.39, *p* < 0.001). Curiously, also in Malaga, participants would pay significantly more for building more dams (mean 3.14, SD 1.04) than in Asturias (mean 2.4, SD 1.1); t = 8.29, *p* < 0.001). The WTP for the rest of actions was not significantly different between regions.

WTP results were coherent with those found for the perception of dam services and impacts in the two regions, Malaga scores being significantly higher than those of Asturias for the two variables (see 3.2). On the one hand, Malaga respondents would pay more for building new dams and reservoirs, thus having more services; on the other, they would pay more for reconnecting the rivers, thus diminishing dam impacts. In the whole survey, the perception of dam services was positively and significantly correlated with the WTP to build more dams (r = 0.44, *p* < 0.001), and the perception of dam impacts with the WTP to reconnect the rivers (r = 0.1, *P* = 0.017) and to remove obsolete dams (r = 0.13, *P* = 0.002).

## Discussion

In this study we demonstrated a higher acceptance of dams in a region more dependent on them for water security than in a wetter region with higher annual precipitation, where dams are less important for water security. This result was likely not biased by gender or education level, that are important psychological variables that may intervene in the acceptance of dams^31^, because the samples were not different for these variables in Asturias and Malaga samples. Our hypothesis regarding dam services was confirmed, since the population of the dry Malaga province perceived more services provision by dams, and this perception mediated positively and significantly the acceptance of dams.

However, opposite to our expectation (ii), perceived dam impacts did not predict significantly their acceptance (or rejection). There is a social consensus about dam impacts that have caused mobilizations against large-scale dams worldwide^35^. Media discourses in Europe emphasize environmental protests in the last decade^36^, thus the majority knows that dams are not harmless. If impacts are a commonplace, a higher or lower degree of perceived impact will not make a difference in the individual acceptance of dams. Lack of predictive value of dam impacts on dam acceptance does not mean that the population does not perceive them; in fact, in Malaga the score of impacts was significantly higher than in Asturias, implying greater awareness about their effects. Benefits will be more important for dam acceptance due to their compensation role. Since inhabitants of Malaga suffer more water shortages than Asturians, they obviously appreciate more the benefits of reservoirs for water supply; but at the same time they can see rivers virtually dry downstream, thus they may perceive the impacts better. In other words, acceptance of dams/reservoirs and recognition of their services was not correlated with unawareness of environmental impacts. This suggests that in dry regions the benefits may compensate the impacts, making dams/reservoirs more accepted (or less rejected). Equal scores for impacts and benefits in Malaga support the idea of compensation to promote dam acceptance. Our results indicate that, although dams and reservoirs have a significant impact on ecosystems and biodiversity^37^, these can be offset in the eyes of the public if the perceived benefits are high^20^.

Environmental values being linked with the preferred policies regarding dam construction^30^, one could ask if the Malaga participants in this study have less ecologically-oriented values than those of Asturias because they would like to build more dams. The answer is likely not, since they would pay also more for actions aimed at the reconnection of rivers. Attitudes are not necessarily linked to knowledge regarding environmental issues^38^, and our results confirm it: Malaga inhabitants are aware of ecological problems caused by dams but their attitude towards them is positive. The idea of compensation of benefits and impacts could be applied here. Throughout their own lifetime, the present generation is an eyewitness of the bioclimatic changes and of the impact that human action causes on ecosystems. Although skeptical scientists still discuss empirical evidence^39,41^, the population is likely becoming aware of living moments of real ecological risk due to climate change, especially in dry regions.

The results of this study are in line with those published in other regions. The majority of the respondents were relatively favorable to dams in the two provinces considered, with means of rejection near or below 2.5 (5 was the maximum). Similarly, the majority of participants in two studies in Poland^25,32^ were favorable to dams. The majority of respondents in a study conducted in Germany, Portugal and Sweden were favorable to hydropower energy for its contribution to mitigate climate change; although with cautions regarding its potential impact on ecosystems and a preference for modernizing current facilities instead of building new ones^42^. Far from Europe, Brazilians displaced by the large Castanhao dam were only marginally unsatisfied ten years after its construction^21^. All together, it seems that, under certain conditions, current dams are relatively accepted in many cases.

Methodologically, this study presents a useful tool for investigating the public opinion about dams and reservoirs that could be applied for adaptive management of river barriers in Europe, and perhaps in other societies with some adaptations to avoid cultural biases. This tool could be improved in several ways. Here, the questionnaire was administered face-to-face. The interviewer's presence and visual availability of the questionnaire in paper or electronic format contributes to the better understanding of the questions, and the questions are presented at a rate adapted to each interviewee, which is satisfactory for the interviewees^43^. In addition to this modality, this questionnaire could also be easily adapted to a self-administered modality^44^, simplifying the work of researchers in data collection and reducing research costs^45^. On the other hand, we used words like dams and reservoirs, suggestive of large impacts. Views of river fragmentation tend to consider only large dams with heights greater than 10 m, ignoring smaller structures. Yet, 85% of barriers in European rivers are small artificial structures such as weirs, ramps, culverts and fords^46^ that are under-represented in barrier inventories^47^. The present study could be adapted to investigate the public perception of small barriers and their impacts, and to assess their acceptance. This would facilitate a more comprehensive understanding of public views about river fragmentation. Other improvements could be to add energy supply to the list of potential benefits in question Q2 (our questionnaire was largely focused on water supply and river connectivity), and to suggest or mention environmental impacts produced during dam construction or in dam’s obsolescence. A limitation of the questionnaire was to place the WTP issue (Q3, see Supplementary Table 1) after the perception of dam benefits (Q2). It is possible that respondents used theoretical tax payments to justify their previous responses. This could be avoided in future studies placing the WTP question before the question about benefits perception.

As a final remark, it is increasingly necessary to consult and involve all actors and stakeholders in decisions about water management^48^^,^^49^. Infrastructures such as dams and reservoirs have historically fulfilled an essential function in water supply and the generation of hydroelectric power. However, scientific progress and a greater environmental awareness emphasize the need to find alternative ways of obtaining drinking water and use other forms of energy generation with a less severe impact on the river's ecosystem. Public concern for environmental problems has been growing over the last 50 years^50^; dam impacts on river ecosystems are a global problem, and society should participate in the search for solutions or at least show their opinion. Measuring the social attitudes of Europeans about dams and reservoirs is essential, and our results suggest that perception of impacts and benefits of such infrastructures depends on regional water security.

From the point of view of adaptive management^27^, the questionnaire employed in our study or a similar one could be used to assess public opinion and help managers make more informed decisions about mitigation alternatives; for example, dam removal versus dam modification. If confirmed in larger samples, the results of our study are significant for policy development and management of water resources in the studied regions. These results suggest that, since Asturians reject dams more and will to build less dams, removal of obsolete and ageing dams might be better accepted in Asturias than in Malaga; while in Malaga options to reconnect the rivers (for example through fish ladders) would be preferred. Malaga inhabitants would pay more to reconnect the rivers, and at the same time they would pay more to build new dams. From these preferences it is implicit that, if really built, new dams should have passages for river biota in order to not fragment the rivers. Briefly, dam demolition policies could be envisaged in Asturias while river management should prioritize measures to reconcile dam presence and biota connectivity in Malaga. These results emphasize the need for managing water resources at local scales. Other policy suggestions for the improvement of water security could be the construction of water conservancy facilities, as well as ecological compensation for different regions—in taxes or otherwise.

## Materials and methods

### Ethics considerations

The competent regional Research Ethics Committee of the Principality of Asturias approved the questionnaire and procedure with the reference 101/16. Following the conduct code of ethical and responsible research^51^, all information collected was anonymous and only used for research purposes. The participants were informed that they could withdraw from the study at any moment. The study aligns with the Declaration of Helsinki.

### Questionnaire

We designed a questionnaire to quantify social attitudes with regard to dams and reservoirs, and to collect demographic and social data (Supplementary table 1). A high score indicates more aversion to dams. Items were organized in three categories:

- Sociodemographic data: gender, age and proximity to a dam/reservoir (classified as users or visitors). Information on educational level and occupation was placed at the end of the questionnaire to reduce respondent discomfort^52^.
- Quantitative scales.
  - Opinion about dams (Q1), with five possible alternative answers scoring 5 to 1 from Q1 in the final version.
  - Elements perceived as benefited (Q2, nine items) or affected (Q4, five items, two of them formulated as positive impacts—for data analysis they are inverted) by dams and reservoirs. Formulated in 1–5 Likert scale.
- Economic valuation of dams/reservoirs (Q3). It included five items whose valuation was based on willingness to pay (WTP) as an increase of annual taxes for improving dams/reservoir services. Likert scale 1–5 according to the percentages specified (0%; 0.1–0.5%; 0.5–1%; 1–5%; higher percentage).

To reduce bias care was taken not to influence the subjects’ answers^53^. However, it is inevitable that other questions may cause interviewees’ to think in depth about dam impacts, and perhaps influence their answers as long as the test advances^54^. Therefore, Q1 question was asked again at the end of the questionnaire. A significant correlation between the first and the last answer would indicate that the design is valid.

### Questionnaire validation

First a panel of experts (N = 10) read and validated the questionnaire content^55^. Second, a pilot survey was conducted using face-to-face method^56^. The researcher approached prospective participants courteously and politely, taking care not to sound intimidating, making demands or putting pressure onto them. After introducing themselves and the AMBER project (https://amber.international/) briefly, researchers informed participants about the objective of the survey. Participants gave consent for their answers to be noted and signed an informed consent form. The questions were read slowly with a clear voice. During the interview the participant was able to read the questions and what the researcher was writing. In the course of the interview the researcher adopted a neutral attitude, not manifesting any opinion or comment that could bias the participant’s answers. At the end of the interview the participant was offered to revise their answers as written by the researcher, to ensure they agreed with the final content.

The participants were a heterogeneous sample (N = 50) of Asturias population and included 54% of interviewees who were frequent users of reservoirs or lived near them. The gender ratio was 1:1. For the age profile, 28% were younger than 30, 54% between 30 and 60 years old and 18% over 60. In the academic level, 32% respondents indicate they had basic studies (only the compulsory studies up to 18 years old), 26% medium academic level (up to two more years of specialized professional training) and 42% higher education.

Interviewees’ occupations were diverse: unemployed, students, homemakers, warehouse assistants, hairdressers, shop assistants, nurses, doctors, teachers of primary and secondary education, clinical psychologists, engineers and so on. Grouped into main sectors, the sample was: industry (2%), services (54%), unemployed (8%), retired (20%), homemakers (10%), students (6%).

Test–retest method was used on a random sample of 10 respondents to measures the test reliability based on its stability taken at two different times. The more similar the samples, the more reliable the test is. Cronbach’s alpha was employed to assess the internal consistency of the questions; values of 0.70 and higher denoting high consistency^57^.

After determining the internal consistency, reliability and construct validity in the pilot survey, a triangulation process^58^^,^^59^ was carried out among the participants of the pilot survey, the panel of experts and the researchers. Participants in the pilot survey were asked to assess the comprehensiveness of each item and the structure of the questionnaire. A panel of experts reviewed the questionnaire again to evaluate both design and final presentation. Researchers collected all suggestions.

### Sampling and characteristics of the regions studied

The questionnaire was administered in two Spanish regions of similar population size with different climates and water security: the provinces of Asturias in the north (1,018,706 inhabitants), and the province of Malaga in the south (1,685,414 inhabitants) (Fig. 2). They are located in the rainy, temperate, Atlantic Arc façade and in the dry, warm, Mediterranean basin respectively. Mean annual precipitation in Asturias between 1981 and 2010 was 1000–1400 mm versus 400–600 mm in Malaga (Fig. 2); we chose that period of time to illustrate the differences between regions because it represents the majority of the lifespan of the participants in the survey, all of them adults. In more recent years the precipitation and temperature were not much different, as we can see in December records of 2012 and 2017 (Table 1). Climate information by province and month can be found in the official page of the Spanish Agency of Meteorology (http://www.aemet.es/es/serviciosclimaticos/vigilancia_clima/resumenes, accessed in February 2022).

The density of river barriers in Asturias is higher than 1 per river kilometer, and between 0.75 and 1 in Malaga^7^. In both provinces, dams are used for drinking water supply (22%), but there are regional differences that illustrate different needs of water security (Table 1). In Malaga 22% of dams are used for irrigation and 29.6% for water transfer to dry areas, while in the rainy Asturias none has these uses; instead they are used for hydropower, industry and flood control (Table 1). Some reservoirs have recreational uses like fishing and kayaking in the two regions (more in Asturias, 13.1%, than in Malaga, 7.4%).

The participants in the study were contacted directly by the researchers in the field near rivers with dams and reservoirs (Nalon River in Asturias and Guadalhorce River in Malaga), or in cities in the proximity of these rivers (Oviedo and Gijón in Asturias, Malaga city in Malaga). Criteria for inclusion were: to be an adult (over 18 in Spain), and resident in the zone for the majority of their life. Persons not meeting the inclusion criteria, and questionnaires that were not revised by the respondent to confirm the answers were excluded from the analysis.

Face-to-face method was employed as described above for the pilot survey. The dam acceptance, impact and services perceived were the constructs considered to test the departure hypothesis. The dependence on dams for water supply was taken as a proxy of water security. Expectedly, respondents from the dry Mediterranean Malaga province in front of the African coast, where droughts are frequent, would appreciate more the services provided by, perceive less impacts of, and be more favorable to dams and reservoirs than those from rainy Asturias. The need of reservoirs for drinking water and agriculture supply is more obvious in the Mediterranean basin.

### Statistics

Standardized Cronbach’s alpha was calculated following De Vellis^60^. Pearson’s correlation coefficient was used in pairwise comparisons, for example to assess the strength of the test–retest correlation on the mean score in the whole test, and the correlation between distractors and standard items. Individual values in the variables Q1 (dam acceptance), Q2 (dam benefits), and Q4 (dam environmental costs) were estimated as the mean of the corresponding items. Multicollinearity was assessed from the variable inflation factor (VIF) as: VIF_*i*_ = 1/(1 − R^2^_*i*_).

Differences between samples for gender ratio and education level distributions were tested employing contingency Chi-square.

A two-way ANOVA was used to assess the differences among indicators of attitude toward dams (three levels corresponding to the three variable constructs i.e. rejection, impacts and services, all in 1–5 Likert scale) and regions (two levels being Malaga and Asturias regions), and Student’s t tests were used for two-group comparisons.

To test the hypothesized mediators between water security and the acceptance of dams, we performed multiple regression analysis, with dam acceptance as dependent variable, and water security, perception of dam services and perception of impacts as independent variables. In this analysis the values assigned to water security were “secure” as 1 (Asturias) or “insecure” as 0 (Malaga), based on their relative levels of precipitation shown in Table 1 and Fig. 2. We used the dummy variable “1” for Asturias (more natural water security) and “0” for Malaga (less natural water security). Mediation is revealed when the predictive value of the independent variable decreases holding the mediator constant, while the mediator should remain a significant predictor of the dependent variable.

Significance threshold was *p* < 0.05, applying Bonferroni correction for multiple tests whenever relevant. Free PAST software^61^ was used for all analysis.

## Supplementary Information

Supplementary Information.

## Data Availability

The raw results i.e. the responses to the questionnaires organized in spreadsheets are available in the *B2share* European repository with https://doi.org/10.23728/b2share.3b8396699a604465b852fd6614b1cc6d and PID: http://hdl.handle.net/11304/ec728f51-bc5b-4a73-9e8b-9ccdd2e28582.

## References

[1] Karr, J.R., & Chu, E.W. Introduction: sustaining living rivers. In *Assessing the Ecological Integrity of Running Waters, Developments in Hydrobiology, *vol 149 (eds. Jungwirth, M., Muhar, S., & S. Schmutz, S.) 1–14. (Springer: Dordrecht, 2000).

[2] Lu S, Dai W, Tang Y, Guo M (2020). A review of the impact of hydropower reservoirs on global climate change. Sci. Total Environ..

[3] Liu C, Ahn CR, An X, Lee SH (2013). Life-cycle assessment of concrete dam construction: comparison of environmental impact of rock-filled and conventional concrete. J. Constr. Eng. Manage..

[4] Maavara T (2020). River dam impacts on biogeochemical cycling. Nat. Rev. Earth Environ..

[5] Grigg NS (2019). Global water infrastructure: state of the art review. Int. J. Water Resour. Dev..

[6] European Environment Agency. *European waters: Assessment of status and pressures 2018*. https://www.eea.europa.eu/publications/state-of-water (Publications Office of the European Union (2018).

[7] Belletti B (2020). More than one million barriers fragment Europe’s rivers. Nature.

[8] Grill G, Lehner B, Lumsdon AE, MacDonald GK, Zarfl C, Liermann CR (2015). An index-based framework for assessing patterns and trends in river fragmentation and flow regulation by global dams at multiple scales. Environ. Res. Lett..

[9] Kim J, An KG (2015). Integrated ecological river health assessments, based on water chemistry, physical habitat quality and biological integrity. Water.

[10] Vörösmarty CJ (2010). Global threats to human water security and river biodiversity. Nature.

[11] McCartney M (2009). Living with dams: managing the environmental impacts. Water Policy.

[12] Van Cappellen P, Maavara T (2016). Rivers in the Anthropocene: global scale modifications of riverine nutrient fluxes by damming. Ecohydrol. Hydrobiol..

[13] Drouineau H, Durif C, Castonguay M, Mateo M, Rochard E, Verreault G, Yokouchi K, Lambert P (2018). Freshwater eels: a symbol of the effects of global change. Fish Fish.

[14] Jones J (2019). A comprehensive assessment of stream fragmentation in Great Britain. Sci. Total Environ..

[15] Reid AJ (2019). Emerging threats and persistent conservation challenges for freshwater biodiversity. Biol. Rev..

[16] Hermoso V, Clavero M, Blanco-Garrido F, Prenda J (2011). Invasive species and habitat degradation in Iberian streams: an analysis of their role in freshwater fish diversity loss. Ecol. Appl..

[17] Maceda-Veiga A (2013). Towards the conservation of freshwater fish: Iberian Rivers as an example of threats and management practices. Rev. Fish Biol. Fish..

[18] Sánchez-Pérez A (2016). Seasonal use of fish passes in a modified Mediterranean river: first insights of the LIFE+ Segura-Riverlink. FiSHMED.

[19] Schiermeir Q (2018). Dam removal restores rivers. Nature.

[20] Benjankar R, Tonina D, McKean JA, Sohrabi M, Chen Q, Vidergar D (2018). Dam operations may improve aquatic habitat and offset negative effects of climate change. J. Environ. Manage..

[21] Tupiño Salinas CE, Pinto Vidal de Oliveira V, Brito L, Ferreira AV, de Araújo JC (2019). Social impacts of a large-dam construction: the case of Castanhão, Brazil. Water Int..

[22] Opperman, J. J. et al. *Valuing Rivers: How the diverse benefits of healthy rivers underpin economies*. WWF Global Science (2018).

[23] Kellner E (2019). Social acceptance of a multi-purpose reservoir in a recently deglaciated landscape in the Swiss Alps. Sustainability.

[24] Boyé, H., & de Vivo, M. The environmental and social acceptability of dams. *Field Actions Sci. Rep.* http://journals.openedition.org/factsreports/4055 (2016).

[25] Wiejaczka Ł, Piróg D, Fidelus-Orzechowska J (2020). Cost-benefit analysis of dam projects: the perspectives of resettled and non-resettled communities. Water Resour. Manag..

[26] Rodeles AA, Galicia D, Miranda R (2017). Recommendations for monitoring freshwater fishes in river restoration plans: a wasted opportunity for assessing impact. Aquat. Conserv..

[27] Birnie-Gauvin K, Tummers JS, Lucas MC, Aarestrup K (2017). Adaptive management in the context of barriers in European freshwater ecosystems. J. Environ. Manag..

[28] Yousefi-Sahzabi A, Unlu-Yucesoy E, Sasaki K, Yuosefi H, Widiatmojo A, Sugaia Y (2017). Turkish challenges for low-carbon society: current status, government policies and social acceptance. Renew. Sustain. Energy Rev..

[29] Jiang H, Lin P, Qiang M (2016). Public-opinion sentiment analysis for large hydro projects. J. Construct. Eng. Manage..

[30] Schulz C, Martin-Ortega J, Glenk K (2019). Understanding public views on a dam construction boom: the role of values. Water Resour. Manage..

[31] Kirchherr J, Pohlner H, Charles KJ (2016). Cleaning up the big muddy: A meta-synthesis of the research on the social impact of dams. Environ. Impact Assess. Rev..

[32] Piróg D, Fidelus-Orzechowska J, Wiejaczka L, Łajczak A (2019). Hierarchy of factors affecting the social perception of dam reservoirs. Environ. Impact Assess. Rev..

[33] Arboleya E, Fernandez S, Clusa L, Dopico E, Garcia-Vazquez E (2021). River connectivity is crucial for safeguarding biodiversity but may be socially overlooked. Insights from Spanish University students. Front. Environ. Sci..

[34] Gilg, A., & Barr, S. Behavioural attitudes towards water saving? Evidence from a study of environmental actions. *Ecol. Econ*. **57**(3), 400–414. doi:10.1016/j.ecolecon.2005.04.010 (2006)

[35] Schapper, A., Unrau, C., & Killoh, S. Social mobilization against large hydroelectric dams: a comparison of Ethiopia, Brazil, and Panama. *Sustain. Develop*. **28**, 413–423. doi:10.1002/sd.1995 (2020)

[36] Flaminio, S., Piégay, H., & Le Lay, Y-F. To dam or not to dam in an age of anthropocene: insights from a genealogy of media discourses. *Anthropocene*. **36**, 100312, doi:10.1016/j.ancene.2021.100312 (2021)

[37] Bellmore JR (2019). Conceptualizing ecological responses to dam removal: If you remove it, what's to come?. Bioscience.

[38] Heberlein TA (2012). Navigating environmental attitudes. Conserv. Biol..

[39] Lewandowsky S, Gignac GE, Vaughan S (2013). The pivotal role of perceived scientific consensus in acceptance of science. Nat. Clim. Change..

[40] Schuldt JP, Roh S, Schwarz N (2015). Questionnaire design effects in climate change surveys: Implications for the partisan divide. Ann. Am. Acad. Pol. Soc. Sci..

[41] Bowden V, Nyberg D, Wright C (2019). Planning for the past: local temporality and the construction of denial in climate change adaptation. Glob. Environ. Change.

[42] Venus, T. E., Hinzmann, M., Bakken, T. H., Gerdes, H., Nunes Godinho, F., Hansen, B., Pinheiro, A., & Sauer, J. The public's perception of run-of-the-river hydropower across Europe. *Energy Policy.* **140**, 111422. doi:10.1016/j.enpol.2020.111422 (2020)

[43] Schober MF (2018). The future of face-to-face interviewing. Qual. Assur. Educ..

[44] Couper MP (2011). The future of modes of data collection. Public Opin. Q..

[45] Zhang X, Kuchinke L, Woud ML, Velten J, Margraf J (2017). Survey method matters: Online/offline questionnaires and face-to-face or telephone interviews differ. Comput. Hum. Behav..

[46] Garcia de Leaniz, C., Berkhuysen, A., & Belletti, B. Beware small dams, they can do damage, too. *Nature* **570**, 164–164; doi:10.1038/d41586-019-01826-y (2019). 10.1038/d41586-019-01826-y 31186570

[47] Belletti, B., et al. Small isn’t beautiful: the impact of small barriers on longitudinal connectivity of European rivers. *Geophys. Res. Abst.* **20**: EGU2018-PREVIEW (2018).

[48] Hophmayer-Tokich S, Krozer Y (2008). Public participation in rural area water management: experiences from the North Sea countries in Europe. Water Int..

[49] San-Martín E, Larraz B, Gallego MS (2020). When the river does not naturally flow: a case study of unsustainable management in the Tagus River (Spain). Water Int..

[50] Dunlap, R. E. *Environmental concern*. The Wiley‐Blackwell Encyclopedia of Globalization. (Wiley, Amsterdam, 2012).

[51] European Commission *Ethics for researchers. Facilitating Research Excellence in FP7*. 10.2777/7491 (Publications Office of the European Union, 2013).

[52] Jenner BM, Myers KC (2019). Intimacy, rapport, and exceptional disclosure: a comparison of in-person and mediated interview contexts. Int. J. Soc. Res. Methodol..

[53] Given LM (2015). 100 questions (and answers) about qualitative research.

[54] Saris WE, Gallhofer IN (2014). Design, evaluation, and analysis of questionnaires for survey research.

[55] Avella JR (2016). Delphi panels: research design, procedures, advantages, and challenges. IJDS.

[56] Vandenplas C, Loosveldt G (2017). Modeling the weekly data collection efficiency of face-to-face surveys: six rounds of the European social survey. J. Surv. Stat. Methodol..

[57] Barbero-García MI, Vila-Abad E, Holgado-Tello FP (2008). Tests adaptation in cross-cultural comparative studies. Acción Psicol..

[58] Flick, U. *Triangulation in data collection*. The SAGE Handbook of Qualitative Data Collection. (Sage, London, 2018).

[59] Heesen R, Bright LK, Zucker A (2019). Vindicating methodological triangulation. Synthese.

[60] DeVellis RF (2012). Scale development: Theory and applications.

[61] Hammer, Ø., Harper, D.A.T., & Ryan, P.D. PAST: paleontological statistics software package for education and data analysis. *Palaeontol. Elect.* **4**(1), 9. http://palaeo-electronica.org/2001_1/past/issue1_01.htm (2001).

