# Supplementary material for: Water security determines social attitudes about dams and reservoirs in South Europe
Source: Sci Rep. 2022 Apr 12;12:6148. doi: 10.1038/s41598-022-10170-7 (PMC9005547; doi:10.1038/s41598-022-10170-7)
Supplement: Supplementary file 1 — Supplementary Information. [file 41598_2022_10170_MOESM1_ESM.docx]

Supplementary Table 1. Final Questionnaire designed for knowing public opinions about dams and reservoirs

***Questionnaire about social attitudes towards dams and reservoirs***

| *** Interviewer:** |  | **Questionnaire nº:** |  |
| --- | --- | --- | --- |

| **Town:** |  | **Country:** |  | **Date:** |  |
| --- | --- | --- | --- | --- | --- |

**If the interview is done on-site, or if the interviewee refers to a particular dam-reservoir-stretch of river:**

| **Dam-reservoir-river area** |  | (please add geographic coordinates if available) |
| --- | --- | --- |

| **Are you neighbor from the area or are you a frequent user of the reservoir?** | **Y** |  | **N** |  |
| --- | --- | --- | --- | --- |

**Personal data:**

| ♂ | ♀ | ***Age*** | - < 30 |  | > 60 |  |
| --- | --- | --- | --- | --- | --- | --- |
|  |  |  | between 30 and 60 |  |  |  |

**Opinion test**

| **Q1) In your opinion, the reservoir/s… (mark with an X)** |  |
| --- | --- |
| 1a) Should be eliminated |  |
| 1b) I would prefer dams and reservoirs out of the area where I’m living |  |
| 1c) Should be modified for having less ecological impact |  |
| 1d) Should be maintained as it is/ they are today |  |
| 1e) More dams and reservoirs are needed to provide more services |  |

| **Q2) In your opinion, how much are the following elements benefited by dams and reservoirs? ___ Between 1 as minimum and 5 as maximum, how much? (0 means ‘I don’t know’ or ‘no answer’)** |  | | | | | |
| --- | --- | --- | --- | --- | --- | --- |
|  | **0** | **1** | **2** | **3** | **4** | **5** |
| 2a) Agriculture and husbandry |  |  |  |  |  |  |
| 2b) Tourism |  |  |  |  |  |  |
| 2c) Fishing (sport or extractive according to country/law ) |  |  |  |  |  |  |
| 2d) Economic development of the region |  |  |  |  |  |  |
| 2e) Historical and industrial heritage |  |  |  |  |  |  |
| 2f) Flood control |  |  |  |  |  |  |
| 2g) Water reserves |  |  |  |  |  |  |
| 2h) Natural environment |  |  |  |  |  |  |
| 2i) Animals and plants |  |  |  |  |  |  |

| **Q3) What percentage (%) of your annual taxes would you allocate to…?** |  | | | | |
| --- | --- | --- | --- | --- | --- |
|  | **0** | **0.1-0.5** | **0.5-1** | **1-5** | **Other** |
| 3a) Building structures to facilitate fish migration |  |  |  |  |  |
| 3b) Improving the economic efficiency of the reservoir (leisure, fishing, water reserves…) |  |  |  |  |  |
| 3c) Improving water connectivity / reconnecting rivers |  |  |  |  |  |
| 3d) Contributing to demolishing dams and reservoirs |  |  |  |  |  |
| 3e) Building new dams and reservoirs |  |  |  |  |  |

| **Q4) Being 1 ‘I totally disagree’ and 5 ‘I completely agree’, you think that reservoirs… (0 means ‘I don’t know’ or ‘no answer’)** |  | | | | | |
| --- | --- | --- | --- | --- | --- | --- |
|  | **0** | **1** | **2** | **3** | **4** | **5** |
| 4a) Benefit the cities |  |  |  |  |  |  |
| 4b) Affect the environment |  |  |  |  |  |  |
| 4c) Block the migration of aquatic species |  |  |  |  |  |  |
| 4d) Are a source of conflicts |  |  |  |  |  |  |
| 4e) Are necessary |  |  |  |  |  |  |

| **Q1.1) After thinking about reservoirs your initial opinions may have changed. Could you please answer the first questions again? In your opinion, the reservoir/s… (mark with an X)** |  |
| --- | --- |
| 1.1a) Should be eliminated |  |
| 1.1b) I would prefer dams and reservoirs out of the area where I’m living |  |
| 1.1c) Should be modified for having less ecological impact |  |
| 1.1d) Should be maintained as it is/ they are today |  |
| 1.1e) More dams and reservoirs are needed to provide more services |  |

**Personal data:**

| **Education level** | Primary |  | Secondary |  | Higher |  |
| --- | --- | --- | --- | --- | --- | --- |

| **Current occupation** |  |
| --- | --- |

***** Thanks for your participation. Following the Responsible Research code of conduct, the information collected is anonymous and will be only employed for this study. After finishing the questionnaire, you can check your answers, confirm or change them. You can withdraw from the study in any moment and in that case this questionnaire will be destroyed. When the results of this investigation are published you will be able to access them.

| **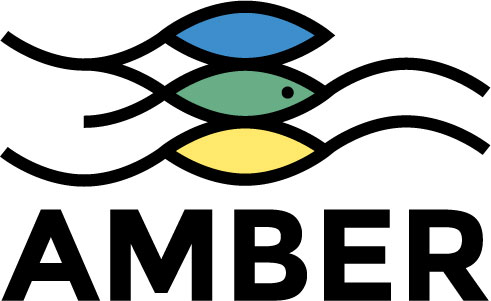** | **UE 16- AMBER-689682**  **Adaptive Management of Barriers in European Rivers** | 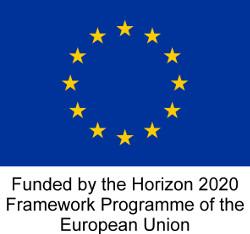 |
| --- | --- | --- |
